# Supplementary material for: Maternal and perinatal obesity induce bronchial obstruction and pulmonary hypertension via IL-6-FoxO1-axis in later life
Source: Nat Commun. 2022 Jul 27;13:4352. doi: 10.1038/s41467-022-31655-z (PMC9329333; doi:10.1038/s41467-022-31655-z)
Supplement: Supplementary file 1 — Supplementary Information [file 41467_2022_31655_MOESM1_ESM.docx]

**Online Supplement**

Maternal and perinatal obesity induce bronchial obstruction and pulmonary hypertension via IL-6-FoxO1-axis in later life

*Jaco Selle^1^, *Katharina Dinger^1,2^, *Vanessa Jentgen^1^, Daniela Zanetti^3,4^, Johannes Will^1^, Theodoros Georgomanolis^5^, Christina Vohlen^1,6,7^, Rebecca Wilke^1^, Baktybek Kojonazarov^7^, Oleksiy Klymenko^7^, Jasmine Mohr^1^, Silke v. Koningsbruggen-Rietschel^8^, Christopher J. Rhodes^9^, Anna Ulrich^9^, Dharmesh Hirani^1,2,7^, Tim Nestler^10^, Margarete Odenthal^2,10^, Esther Mahabir^11^, Sreenath Nayakanti^12^, Swati Dabral^12^, Thomas Wunderlich^2,13,14^, James Priest^3^, Werner Seeger^7,12,15^, Jörg Dötsch^6^, Soni S. Pullamsetti^7,12,15^, Miguel A. Alejandre Alcazar^1,2,7,14,15^

*These authors contributed equally.

^1^University of Cologne, Faculty of Medicine and University Hospital Cologne, Translational Experimental Pediatrics - Experimental Pulmonology, Department of Pediatric and Adolescent Medicine, Germany; ^2^University of Cologne, Faculty of Medicine and University Hospital Cologne, Center for Molecular Medicine Cologne (CMMC), Germany; ^3^Division of Cardiovascular Medicine, Department of Medicine, Stanford University School of Medicine, Stanford, California, USA; ^4^Stanford Cardiovascular Institute, Stanford University, Stanford, California, USA; ^5^University of Cologne, Faculty of Medicine and University Hospital Cologne, Cologne Center for Genomics (CCG); ^6^University of Cologne, Faculty of Medicine and University Hospital Cologne, Department of Pediatric and Adolescent Medicine, Germany; ^7^Institute for Lung Health (ILH), University of Giessen and Marburg Lung Centre (UGMLC), Member of the German Centre for Lung Research (DZL), Gießen, Germany; ^8^University of Cologne, Faculty of Medicine and University Hospital Cologne, Pediatric Pulmonology, Department of Pediatric and Adolescent Medicine, Germany; ^9^National Heart and Lung Institute, Hammersmith Campus, Imperial College London, London, United Kingdom; ^10^University of Cologne, Faculty of Medicine and University Hospital Cologne, Institute of Pathology; ^11^University of Cologne, Faculty of Medicine and University Hospital Cologne, Comparative Medicine, Center for Molecular Medicine Cologne (CMMC), Germany; ^12^Department of Lung Development and Remodeling, Max-Planck-Institute for Heart and Lung Research, Member of the German Center for Lung Research (DZL), Bad Nauheim, Germany; ^13^Max-Planck-Institute for Metabolism Research, Cologne, Germany ^14^Cologne Excellence Cluster for Stress Responses in Ageing-Associated Diseases (CECAD); ^15^Department of Internal Medicine, German Center for Lung Research (DZL), Cardio-Pulmonary Institute (CPI), Justus Liebig University, Giessen, Germany.

**Supplementary Figure 1**


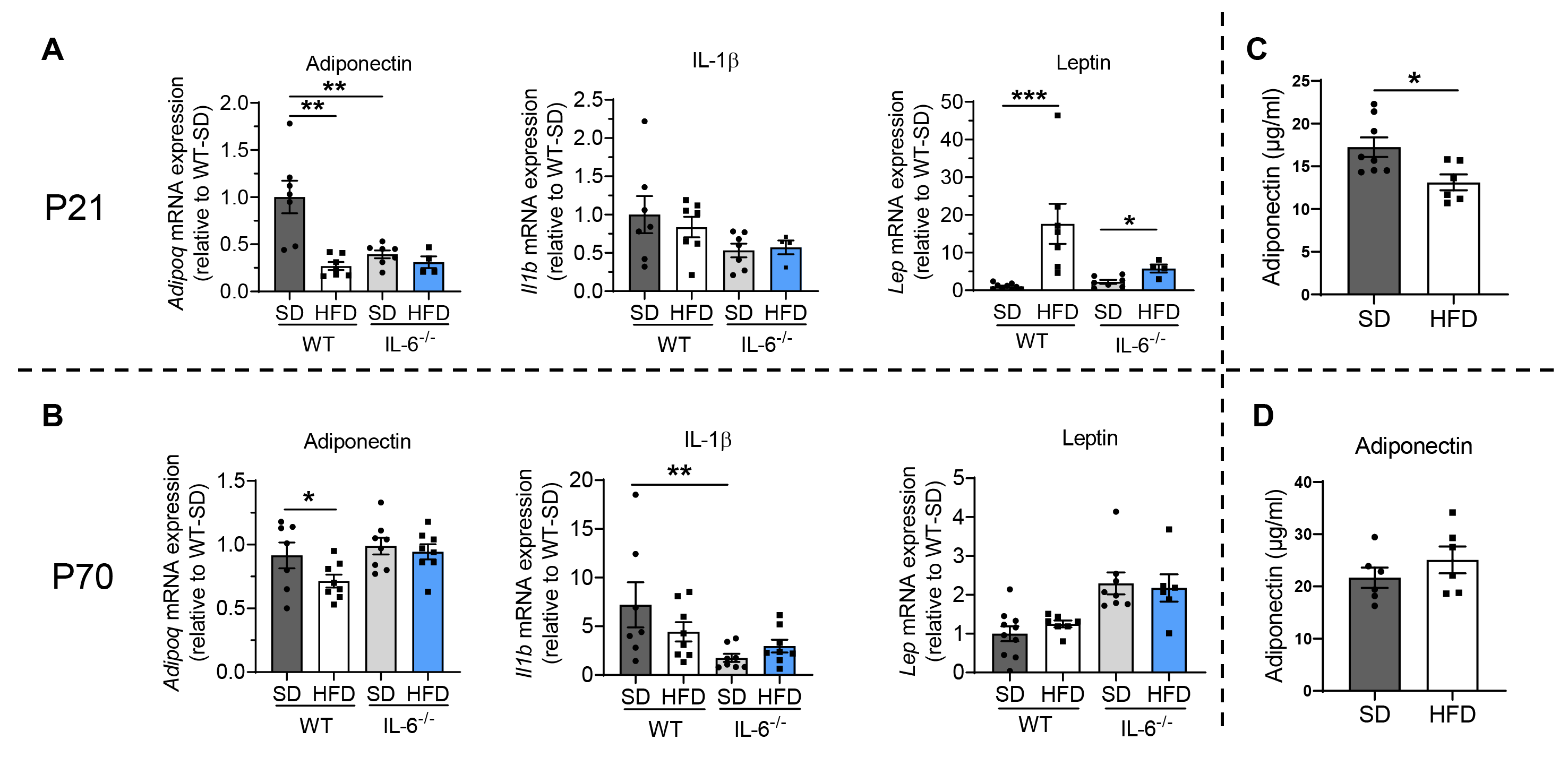


**Supplementary figure 1**. A, B: Expression genes encoding adipocytokines adiponectin (*Adipoq*) interleukin 1β (*Il1b*), and leptin (*Lep*) in epigonadal white adipose tissue (WAT) from wildtype (WT) and IL-6^-/-^ mice of high fat diet (HFD)- or standard diet (SD)-fed dams using qRT-PCR at postnatal day 21 (P21) (A) and P70 (B). C, D: Measurement of serum concentration of adiponectin at P21 (C) and P70 (D) using ELISA. Data are shown as mean ± standard error of the mean. A: WT^SD^ n=7, WT^HFD^ n=7; IL-6^-/-^ ^SD^ n=7, IL-6^-/-^ ^HFD^ n=4, p=0.0014 and 0.0051; WT^SD^ n=7, WT^HFD^ n=7; IL-6^-/-^ ^SD^ n=7, IL-6^-/-^ ^HFD^ n=4; WT^SD^ n=9, WT^HFD^, n=7; IL-6^-/-^ ^SD^ n=7, IL-6^-/-^ ^HFD^ n=4, p=0.0002 and 0.0242; B: WT^SD^ n=6, WT^HFD^ n=8; IL-6^-/- SD^ n=8, IL-6^-/- HFD^ n=8, p=0.0316; WT^SD^ n=7, WT^HFD^, n=8; IL-6^-/-^ ^SD^ n=8, IL-6^-/-^ ^HFD^, n=8; p=0.0090; WT^SD^ n=10, WT^HFD^ n=7; IL-6^-/-^ ^SD^ n=8, IL-6^-/-^ ^HFD^ n=6, C: WT^SD^ n=8, WT^HFD^ n=5, p=0.0426; D: WT^SD^ n=6, WT^HFD^ n=6. Data were analyzed using the two-sided Mann-Whitney test; *p<0.05; **p<0.01; ***p<0.001. Dark grey=wildtype (WT) standard diet (SD); White=WT high-fat diet (HFD); light grey=IL-6^-/- SD^, blue=IL-6^-/- HFD^. Source data are provided in the Supplementary Source Data file.

**Supplementary Figure 2**


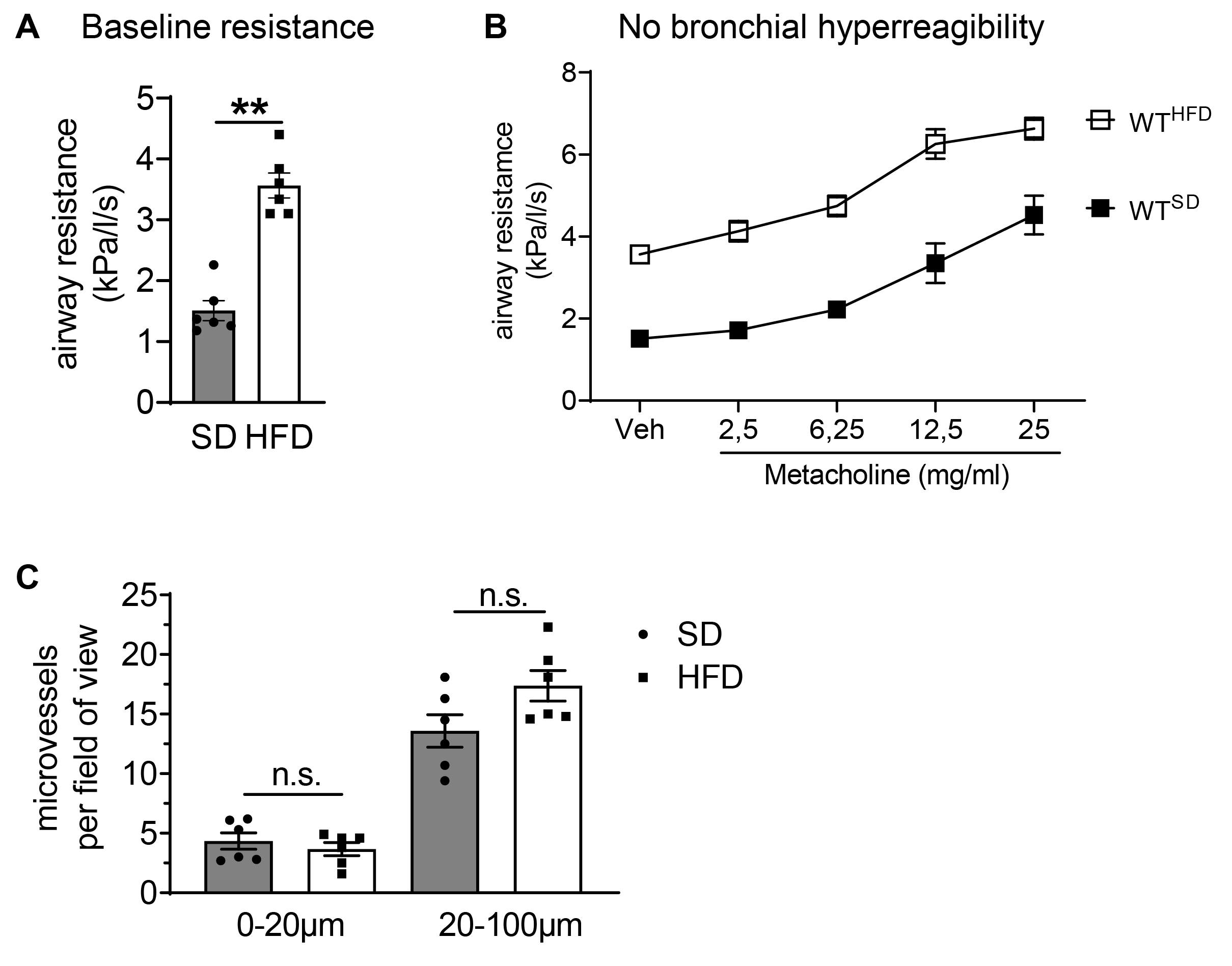


**Supplementary figure 2.** A, B: Airway resistance at baseline (A) and bronchial hyperreagibility (B) were measured in wildtype (WT) offspring of high fat diet (HFD)- or standard diet (SD)-fed dams using body plethysmography at postnatal day 70 (P70). Mice received nebulized PBS at baseline, followed by increasing doses of methacholine (2.5, 6.25, 12.5, and 25 mg/ml). C: Microvessels per field of view (0-20 µm, 20-100 µm) were assessed in lungs at P70 (20x magnification). Data are shown as mean ± standard error of the mean. A: SD n=6, HFD n=6, p=0.0022; B: SD n=6, HFD n=6; C: SD n=6, HFD n=6. Data were analyzed using the two-sided Mann-Whitney test; **p<0.01. Grey=standard diet; White=high-fat diet. Source data are provided in the Supplementary Source Data file.

**Supplementary Figure 3**

**Supplementary figure 3**. A: Representative images of lungs stained for immune cell markers from wildtype (WT) offspring of high fat diet (HFD)- or standard diet (SD)-fed dams: CD3 (T lymphocytes), CD45 (pan-leucocyte marker), and Ly6G (predominantly neutrophils). B-C: Quantification was performed when immune cells were detectable: CD3^+^ cells at postnatal day 21 (P21) and P70 (B), CD45^+^ cells at P70 (C); CD45^+^ cells were not detectable at P21, and Ly6G^+^ cells at P21 and P70; 6-10 fields of view per section, 1 section per animal. Data are shown as mean ± standard error of the mean. B: SD n=5, HFD n=5; SD n=5, HFD n=5; C: SD n=6, HFD n=6. Data were analyzed using the two-sided Student’s t-test. Grey=standard diet; White=high-fat diet. Source data are provided in the Supplementary Source Data file.


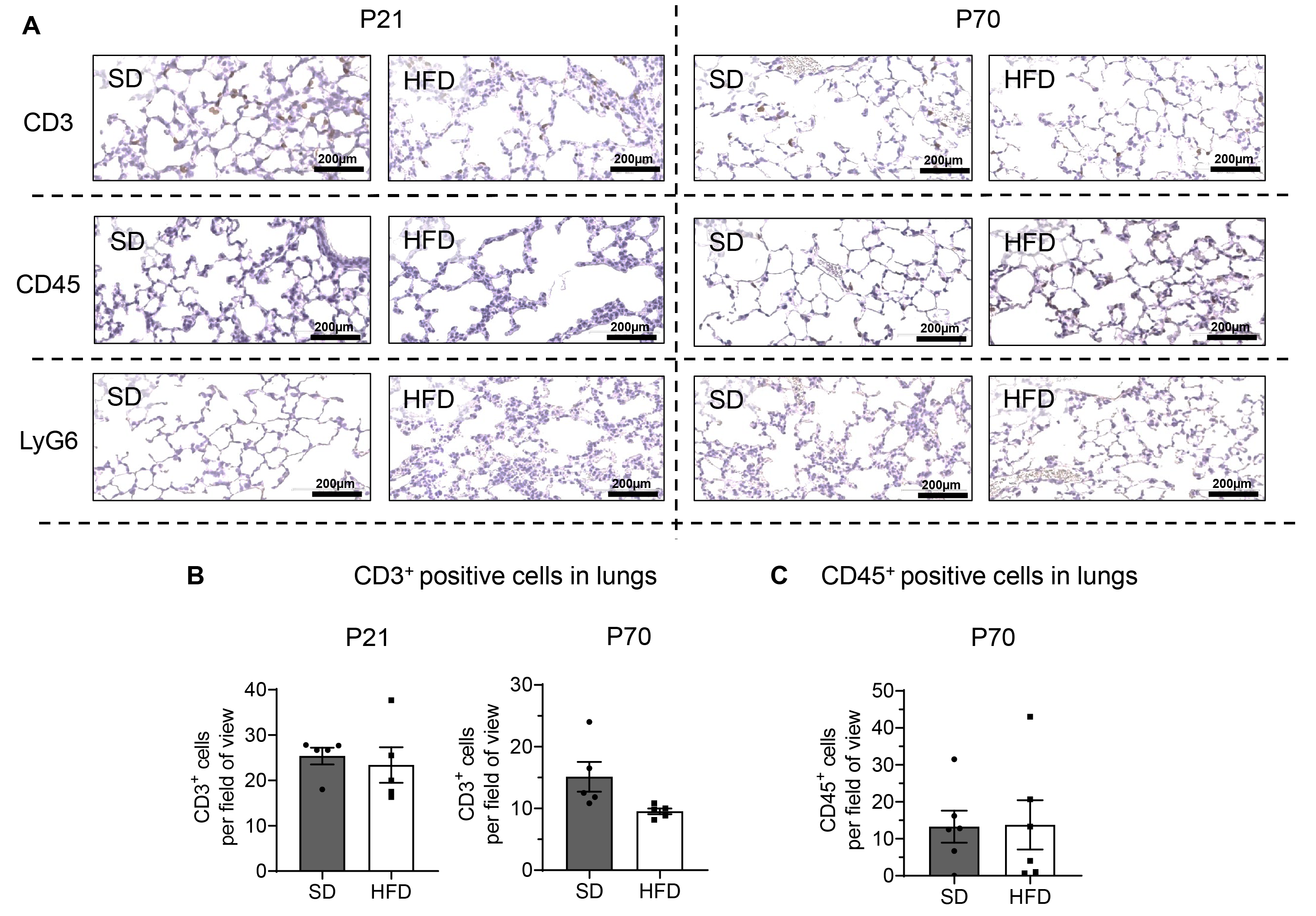


**Supplementary Figure 4**


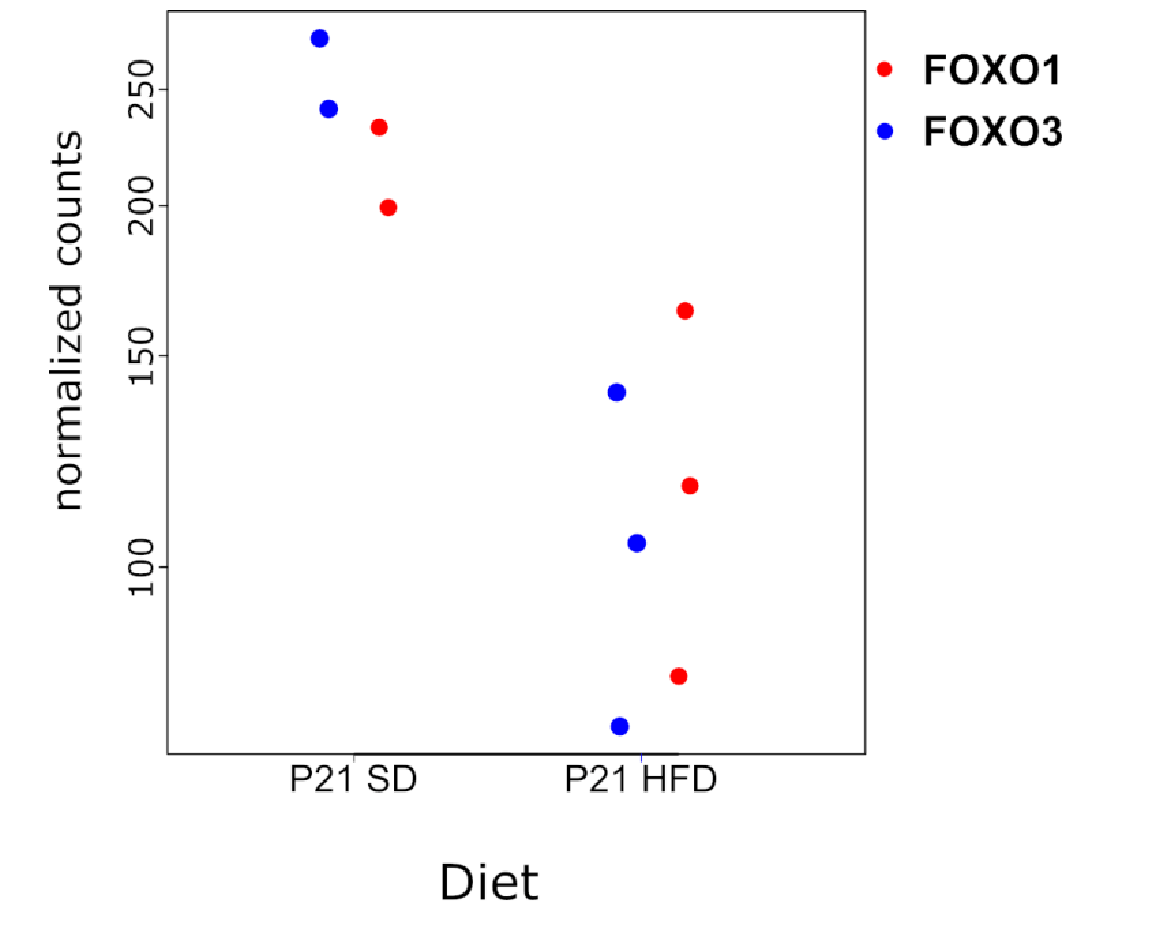


**Supplementary figure 4**. Transcriptomic analysis of laser-microdissected bronchi and vessels from the lungs of the offspring of dams fed a high-fat diet (HFD) or control dams fed a standard diet (SD) at postnatal 21. The DESeq2 normalized counts of *Foxo1* and *Foxo3* are shown.

**Supplementary Figure 5**


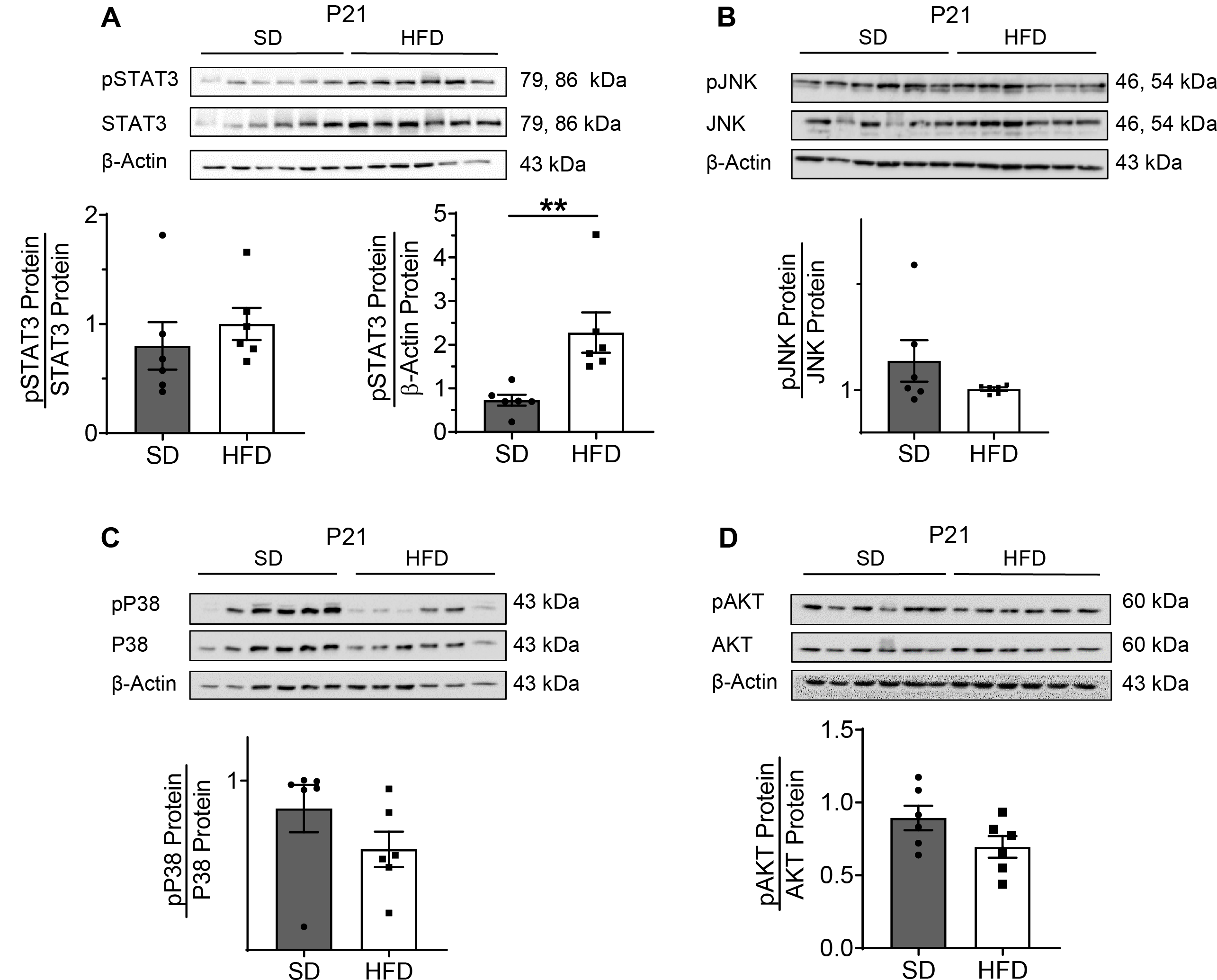


**Supplementary figure 5.** A-D: Assessment of phosphorylated STAT3 (pSTAT3) and total STAT3 (A), pJNK and total JNK (B), pP38 and total P38 (C) as well as pAKT and total AKT (D) in total lung homogenates of wildtype offspring of high fat diet (HFD)- or standard diet (SD)-fed dams using immunoblot at postnatal day 21 (P21); β-actin served as a loading control. A densitometric summary of the pSTAT3, pJNK, pP38, and pAKT data relative to total STAT3 (and β-actin), total JNK, total P38, and total AKT, respectively, is displayed under the immunoblot. Data are shown as mean ± standard error of the mean. A: SD n=6, HFD n=6; SD n=6, HFD n=6; p=0.0090; B: SD n=6, HFD n=6; C: SD n=6, HFD n=6; D: SD n=6, HFD n=6. Data were analyzed using the two-sided Mann-Whitney test. **p<0.01. Grey=standard diet; White=high-fat diet. Source data are provided in the Supplementary Source Data file.

**Supplementary Figure 6**


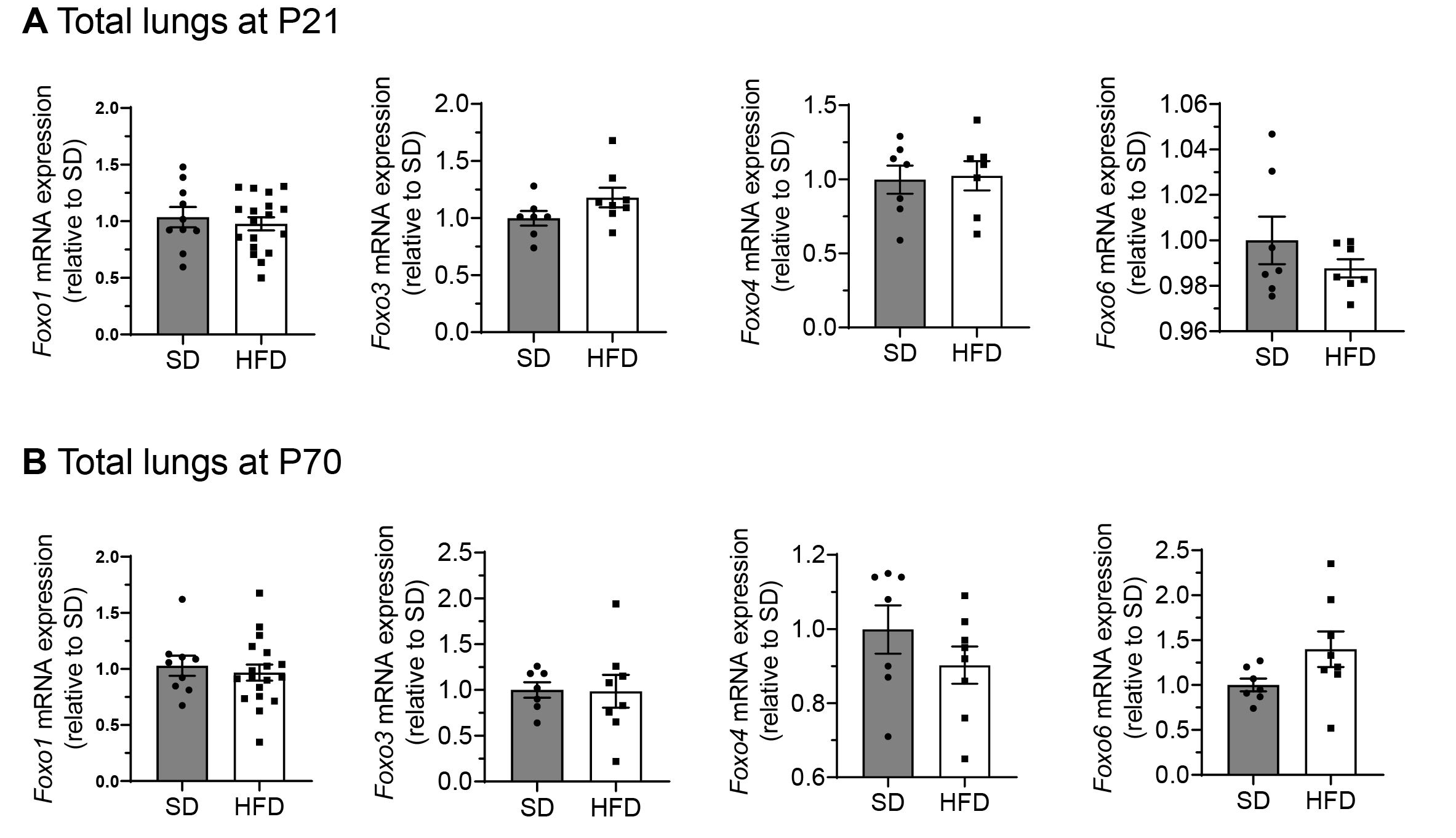


**Supplementary figure 6**. A, B: Expression of genes encoding *Foxo1,* *Foxo3*, *Foxo4*, and *Foxo6* in total lung homogenates from wildtype (WT) mice of high fat diet (HFD)- or standard diet (SD)-fed dams using qRT-PCR at postnatal day 21 (P21) (A) and P70 (B). Data are shown as mean ± standard error of the mean. A: SD n=10, HFD n=18; SD n=7, HFD n=8; SD n=7, HFD n=7; SD n=7, HFD n=7; B: SD n=9, HFD n=18; SD n=7, HFD n=8; SD n=7, HFD n=8; SD n=7 HFD n=8. Data were analyzed using the two-sided Mann-Whitney test. black=standard diet; White=high-fat diet. Source data are provided in the Supplementary Source Data file.

**Supplementary Figure 7**


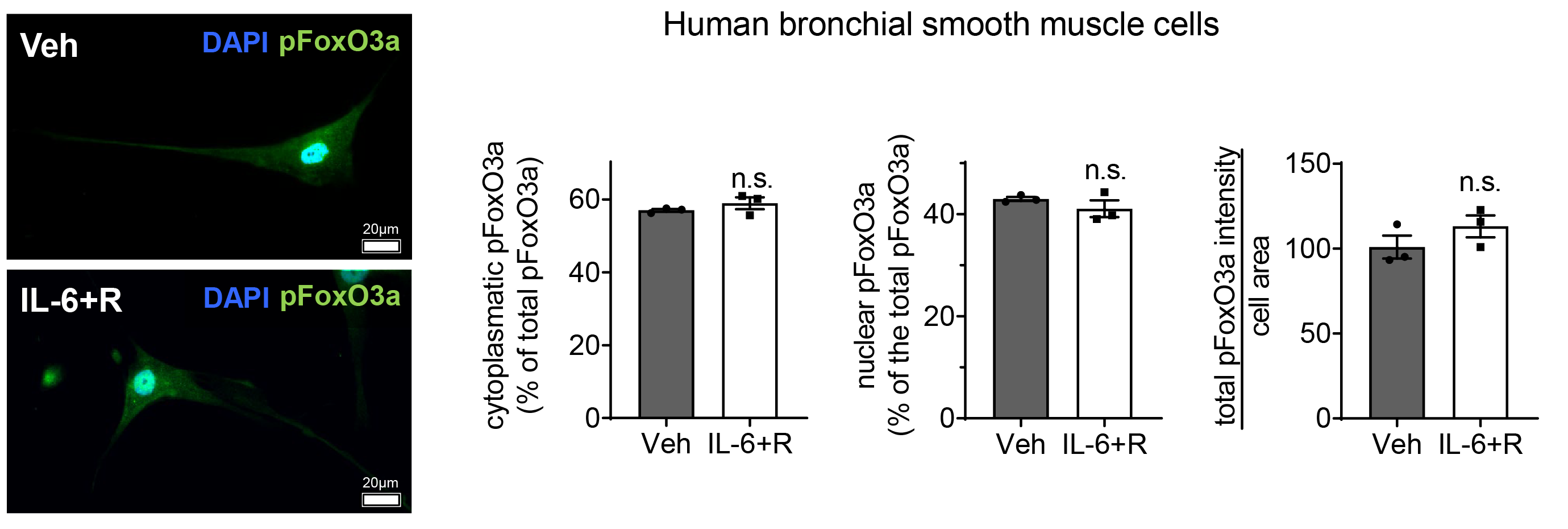


**Supplementary figure 7**. Representative immunofluorescent staining for pFoxO3a (green) and DAPI (nucleus) of human bronchial smooth muscle cells after exposure to IL-6+R (100ng/ml IL-6 and 20ng/ml sIL-6-receptor) or vehicle (0.1% BSA) in serum-reduced medium for 30 min. Quantification of the nuclear and cytoplasmatic phosphorylated FoxO3a (pFoxO3a) fraction in % of total cell pFoxO3a per bSMC; Total pFoxO3a and DAPI per cell area. Data are shown as mean ± standard errors of the mean. A: SD n=3, HFD n=3; SD n=3, HFD n=3; SD n=3, HFD n=3. Data were analyzed using the two-sided Mann-Whitney test. Grey=standard diet; White=high-fat diet. Source data are provided in the Supplementary Source Data file.

**Supplementary Figure 8**


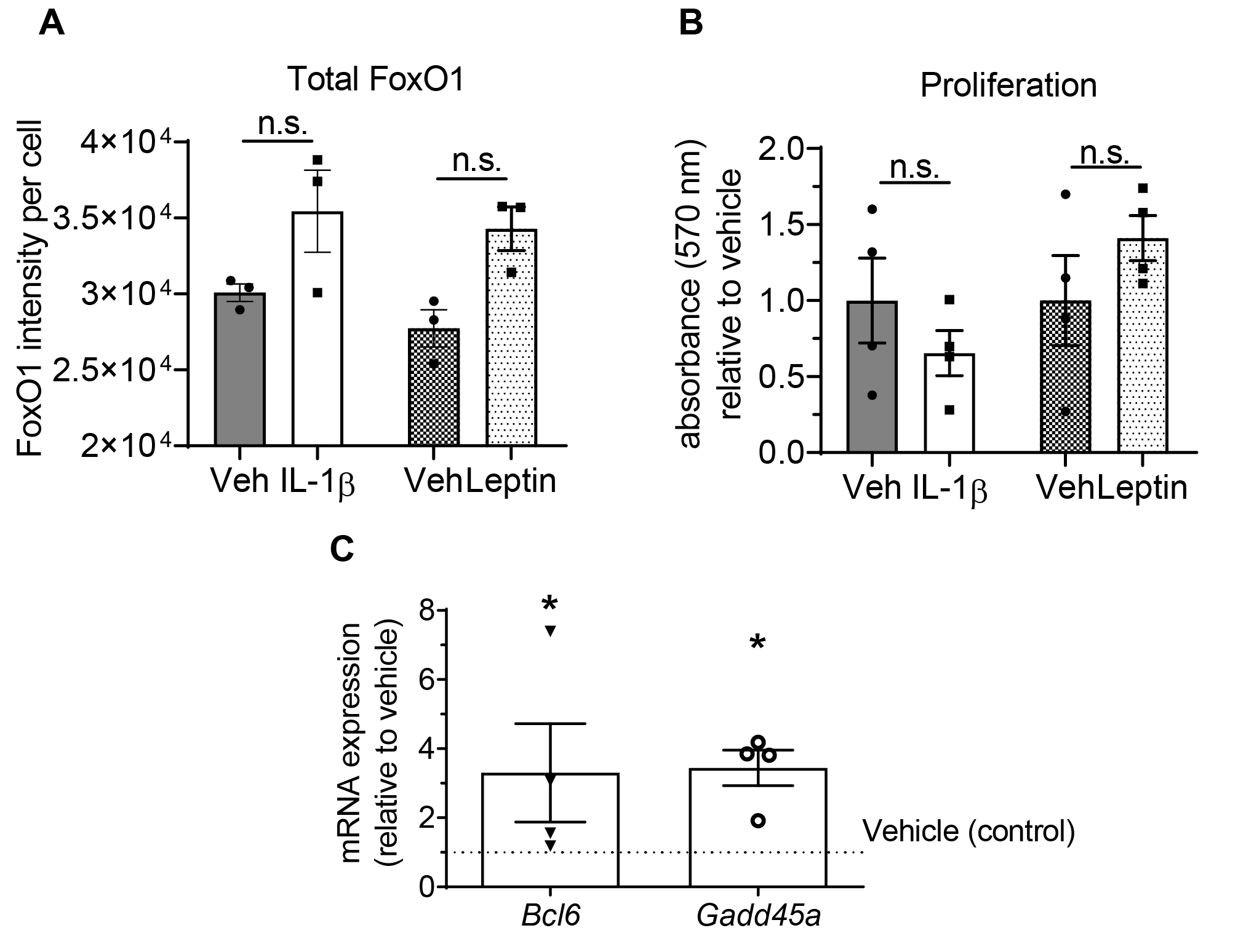


**Supplementary figure 8.** A, B: murine bronchial smooth muscle cell (bSMC) were treated with IL-1β (10 ng/ml, vehicle: aqua dest) or leptin (100 ng/ml, vehicle: 20 mM Tris-HCL, pH 8.0) followed by immuncytochemistry for total FoxO1 and DAPI (nucleus) after 24 h. Quantification of total FoxO1 per cell using FoxO1 intensity (A). Assessment of proliferation (BrdU) after exposure to IL-1β or leptin and their respective vehicles for 24 h (B). C: assessment of *Bcl6* and *Gadd45a* mRNA expression using qRT-PCR in primary bronchial SMCs exposed to Paclitaxel (1µM) or vehicle (ethanol); the vehicle (control) is set to 1. Data are shown as mean ± standard error of the mean. A: Vehicle n=3, IL-1ß n=3; Vehicle n=3, Leptin n=3; B: Vehicle n=4, IL-1ß n=4; Vehicle n=4, Leptin n=4; C: Vehicle n=4, Bcl6 n=4, Gadd45a n=4; p=0.0286; 0.0286. Data were analyzed using the two-sided Mann-Whitney test; n.s. = not significant; *p<0.05. Grey=standard diet; White=high-fat diet. Source data are provided in the Supplementary Source Data file.

**Supplementary Figure 9**


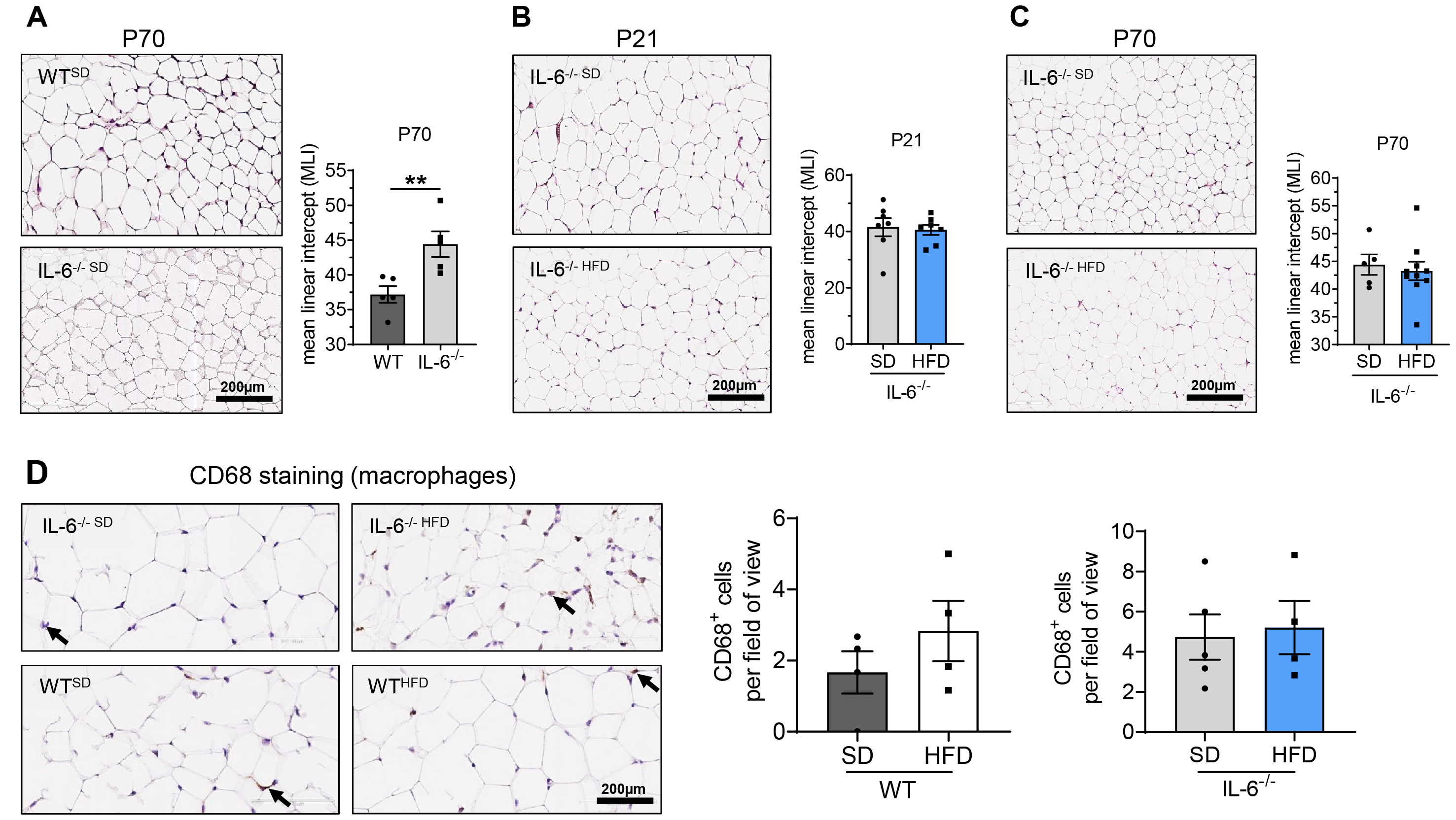


**Supplementary figure 9.** A-C: Quantitative histomorphometric analyses of adipocyte size using mean linear intercept (MLI) as a parameter. MLI was assessed in epigonadal white adipose tissue (WAT) of wildtype (C57BL/6J) and IL-6^-/-^ mice at postnatal day 70 (P70) (A). The effect of maternal and perinatal high fat diet (HFD) on adipocyte size compared to standard diet (SD) was assessed in epigonadal WAT of IL-6^-/-^ mice at P21 (B) and P70 (C). D: Representative images of epigonadal WAT of WT and IL-6^-/-^ offspring of HFD- and SD-fed dams stained for CD68 as a marker of macrophages at P70. The number of CD68^+^ cells per field of view is shown in the graphs next to the respective images. Representative images were taken with 20x magnification. Data are shown as mean ± standard errors of the mean. A: WT^SD^ n=5, IL-6^-/- SD^ n=5; p=0.0079, B: IL-6^-/- SD^ n=7, IL-6^-/- HFD^ n=7; C: IL-6^-/- SD^, n=5 IL-6^-/- HFD^ n=10; D: WT^SD^ n=4, WT^HFD^ n=4; IL-6^-/- SD^ n=5; IL-6^-/- HFD^ n=4. Data were analyzed using the two-sided Mann-Whitney test; **p<0.01. Grey=standard diet; White=high-fat diet; blue=IL-6^-/-^. Source data are provided in the Supplementary Source Data file.

**Supplementary Figure 10**


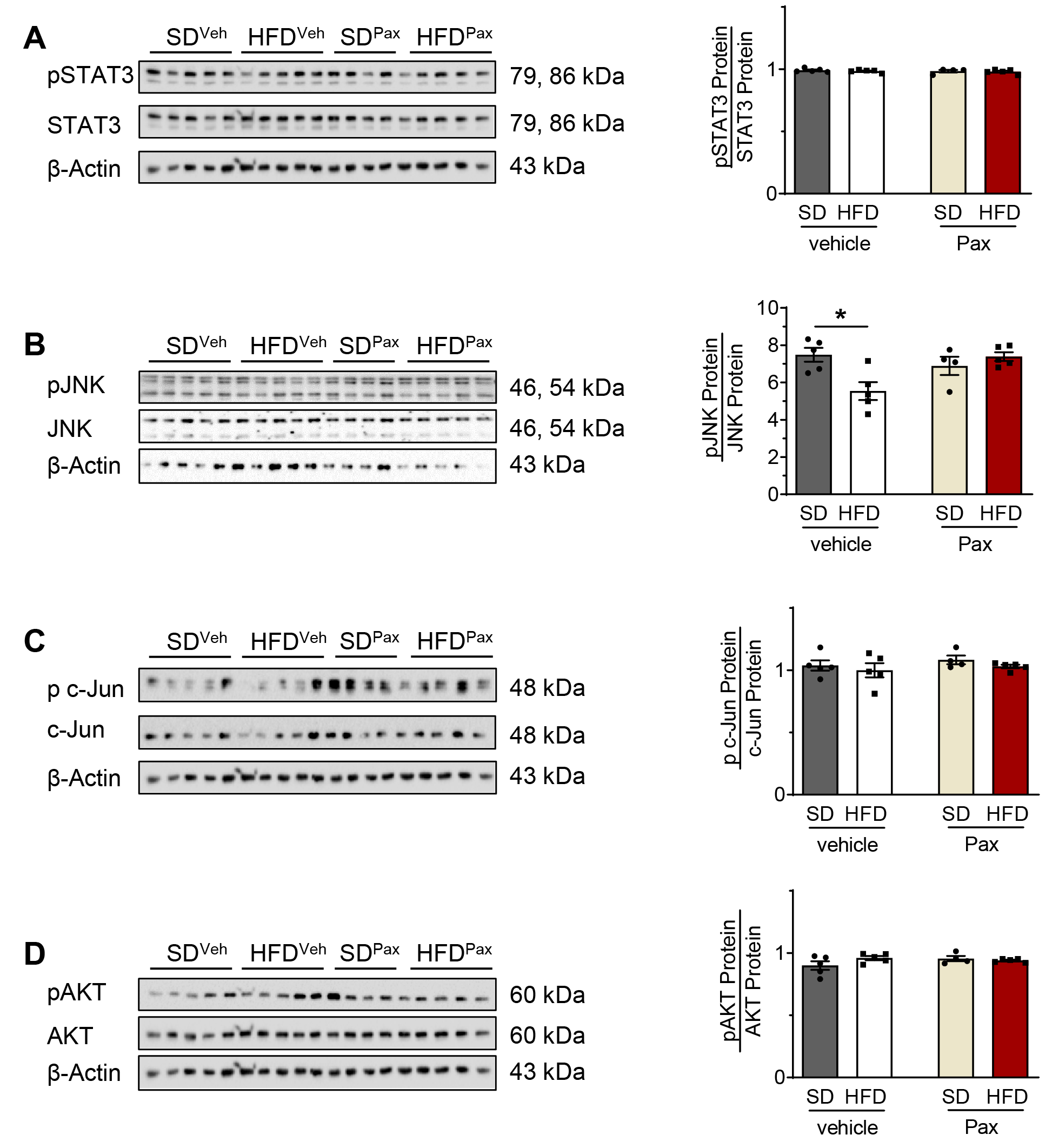


**Supplementary figure 10.** A-D: Immunoblots of phosphorylated STAT3 (pSTAT3) and total STAT3 (A), pJNK1 and total JNK1 (B), pc-Jun and total c-Jun (C) as well as pAKT and total AKT (D) in total lung homogenates of wildtype offspring of high fat diet (HFD)- or standard diet (SD)-fed dams at postnatal day 70 (P70). Offspring were treated with Paclitaxel or vehicle i.v.; β-actin served as a loading control. A densitometric summary of the pSTAT3, pJNK, p c-Jun, and pAKT data relative to total STAT3, total JNK, total c-Jun and total AKT, respectively, is displayed next to the immunoblot. Data are shown as mean ± standard error of the mean. A: SD n=5, HFD n=5; SD n=4, HFD n=5; B: SD n=5, HFD n=5; SD n=4, HFD n=5; p=0.0125; C: SD n=5, HFD n=5; SD n=4, HFD n=5; D: SD n=5, HFD n=5; SD n=4, HFD n=5. Data were analyzed using the two-sided Mann-Whitney test; *p<0.05. Grey=standard diet; White=high-fat diet; Yellowish=standard diet+Paclitaxel; Reddish=high-fat diet+Paclitaxel. Source data are provided in the Supplementary Source Data file.

**Supplementary Figure 11**


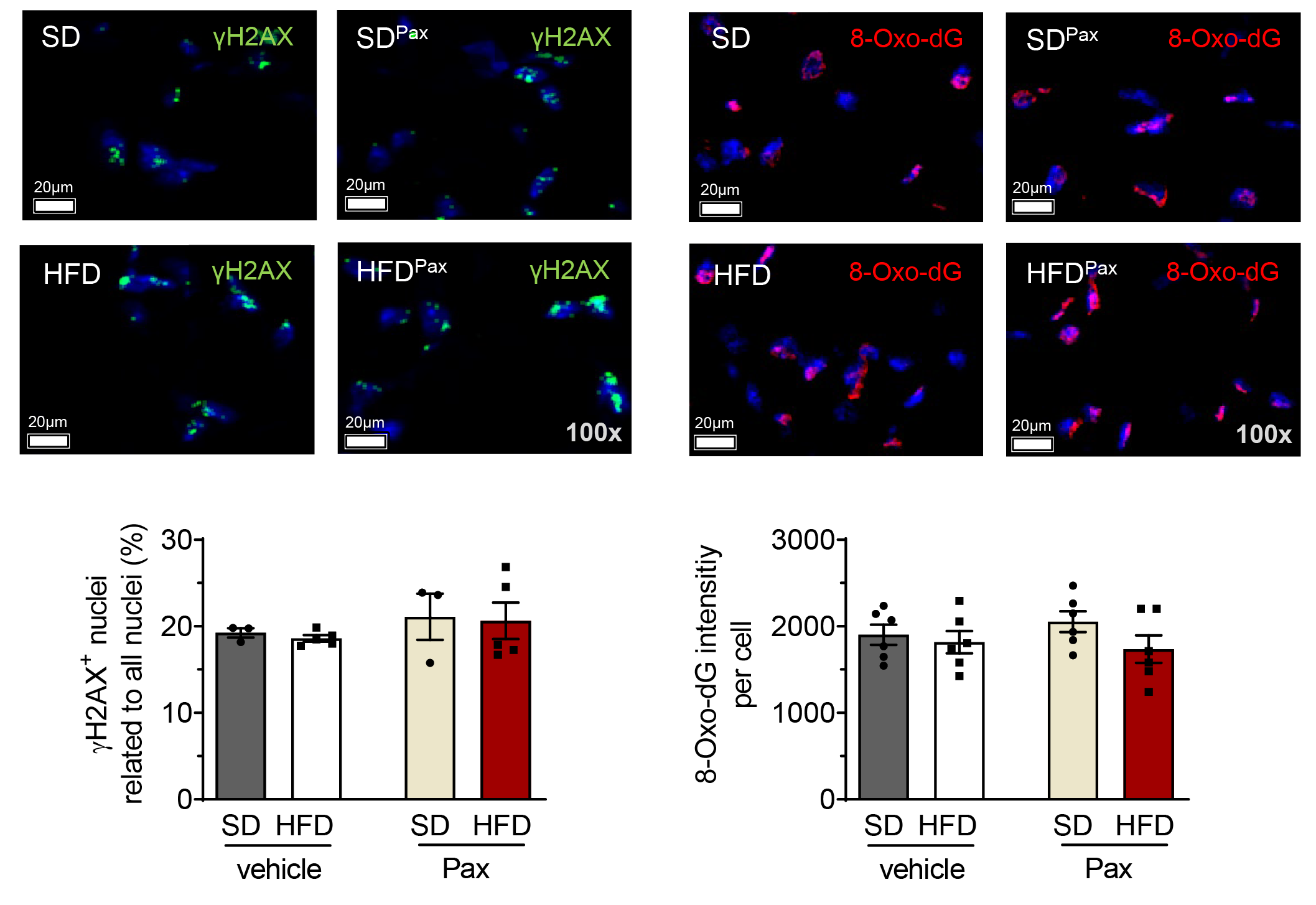


**Supplementary figure 11.** Representative images of lungs stained for γH2AX (green) or 8-Oxo-2’-deoxyganosine (8 Oxo-dG, red) as an indicator of DNA damage and oxidative damage to DNA, respectively, and DAPI (blue, nucleus). Assessment of the percentage of γH2AX^+^ cells relative to all nuclei and 8 Oxo-dG intensity per cell in lungs of wildtype offspring of high fat diet (HFD)- or standard diet (SD)-fed dams at postnatal day 70 (P70). Offspring were treated with Paclitaxel or vehicle i.v. at postnatal day 50 (P50); wildtype offspring of high fat diet (HFD)- or standard diet (SD)-fed dams. Data are shown as mean ± standard error of the mean. A: SD n=3, HFD n=5; SD n=3, HFD n=5; B: SD n=6, HFD n=6; SD n=6, HFD n=6. Data were analyzed using the two-sided Mann-Whitney test. Grey=standard diet; White=high-fat diet; Beige=standard diet+Paclitaxel; Red=high-fat diet+Paclitaxel. Source data are provided in the Supplementary Source Data file.

**Supplementary Figure 12**


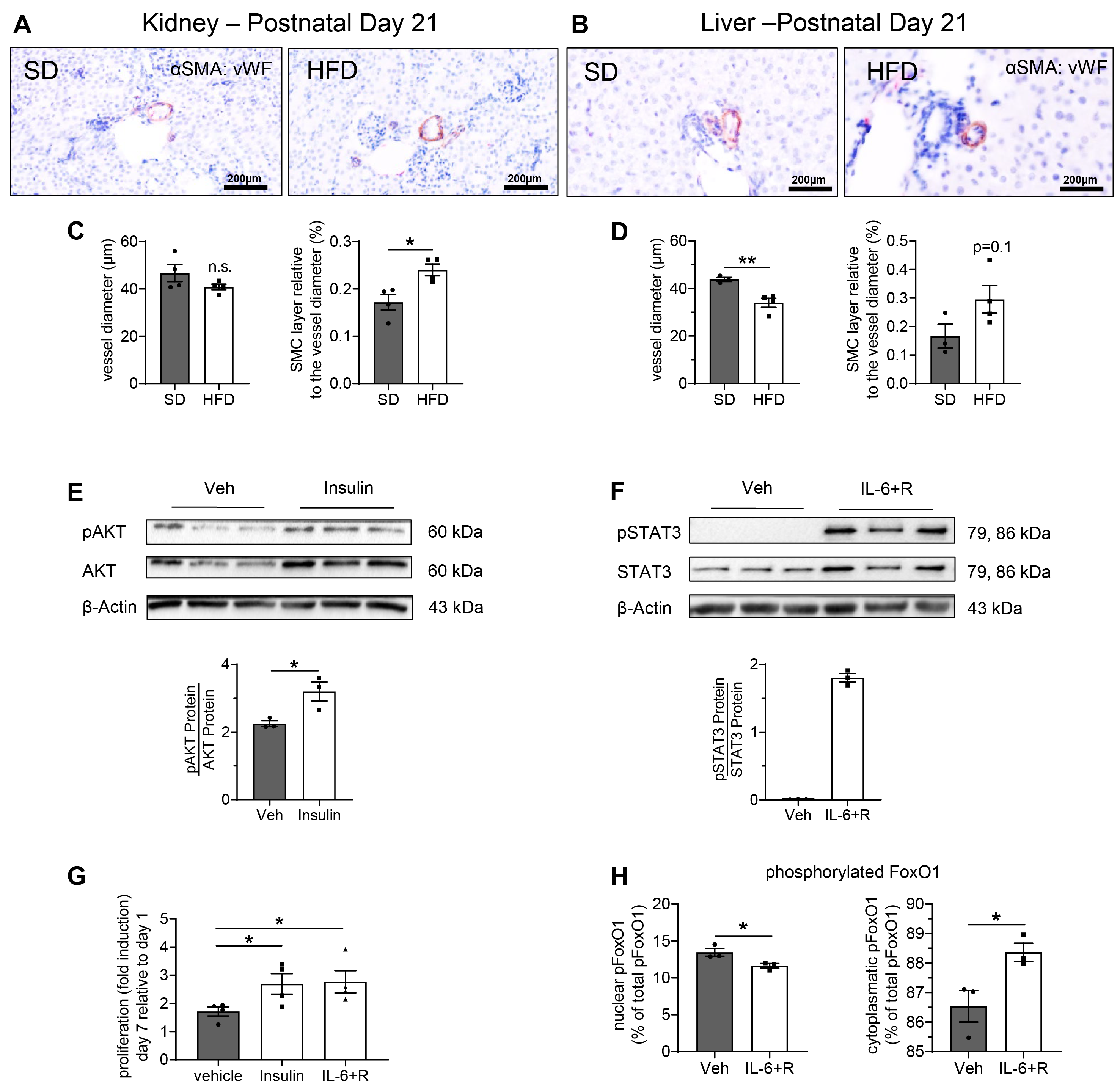


**Supplementary figure 12.** A, B: Representative images displaying vascular staining in kidneys (A) and liver (B) of offspring of high-fat diet- (HFD) of standard diet- (SD) fed dams at postnatal day 21 (P21). Immunhistochemical staining was performed against α smooth muscle actin (αSMA) and von Willebrand factor (vWF) as markers of smooth muscle cells (SMCs) and endothelial cells, respectively. C, D: In random kidney (C) and liver sections (D), we assessed the average diameter of the vessels and SMC layer thickness relative to the vessel diameter. E, F: Immunoblots showing phosphorylated AKT (pAKT) and total AKT (E) as well as pSTAT3 and total STAT3 (F) in human aortic SMCs (haSMC) after exposure to insulin (10µg/ml) or Interleukin 6 (IL-6) with soluble IL-6 receptor (IL-6+R; 100ng/ml and 20ng/ml) 30 min; β-actin was used as a loading control. A densitometric summary of the pAKT and pSTAT3 data relative to total AKT and total STAT3, respectively, is displayed below the immunoblot. G: Assessment of proliferation (cell count) over seven days after exposure to insulin, IL-6+R or the respective vehicle. H: Quantification of the nuclear and cytoplasmatic phosphorylated FoxO1 (pFoxO1) fraction in % of total cell pFoxO1 per haSMC using immunofluorescent staining for pFoxO1 and DAPI; haSMC were treated with IL-6+R for 30 min.Data are shown as mean ± standard error of the mean. C: SD n=4, HFD n=4; SD n=4, HFD n=4; p=0.0157; D: SD n=3, HFD n=4; SD n=3, HFD n=4; p=0.0091; E: Vehicle n=3, Insulin n=3; p=0.0319; F: Vehicle n=3, IL-6 n=3; G: Vehicle n=4, Insulin n=4, IL-6 n=4; p=0.0482; 0.0483, H: Vehicle n=3, IL-6 n=3; p=0.0409; Vehicle n=3, IL-6 n=3, p=0.0409. Data were analyzed using the two-sided Student’s t-test.. *p<0.05; **p<0.01. Grey=standard diet/vehicle; White=high-fat diet/stimulation. Source data are provided in the Supplementary Source Data file.

Supplementary Table 1

Primer-sequences for qPCR analysis

| Taqman primer | | |
| --- | --- | --- |
| *Acta2* | forward | ACATCAGGGAGTAATGGTTGGAAT |
|  | reverse | GGTGCCAGATCTTTTCCATGTC |
|  | taq | CGATAGAACACGGCATCATCACCAACTG |
| *Foxo1* | forward | GCGGGCTGGAAGAATTCAA |
|  | reverse | TGAGCATCCACCAAGAACTCTTT |
|  | taq | TCGCCACAATCTGTCCCTTCACAGC |
| *Ccnb1* | forward | GTAACGGCCATGTTTATTGCAA |
|  | reverse | TCTGGTGCTTAGTGTACGTGTTGTT |
|  | taq | TGTACCCTCCAGAAATAGGTGACTTCGCCT |
| *Cdkn1b* | forward | CAAAAGGGCCAACAGAACAGA |
|  | reverse | CGAAGGCCGGGCTTCTT |
|  | taq | AAAATGTTTCAGACGGTTCCCCGAACG |
| *bcl6* | forward | GGCCTCCTTCCGCTACAAG |
|  | reverse | TGCGCTCCACAAATGTTACAG |
|  | taq | CCACAAGACTGTCCACACGGGTGAGA |
| *Il6* | forward | ACAAGTCGGAGGCTTAATTACACAT |
|  | reverse | AATCAGAATTGCCATTGCACAA |
|  | taq | TCTTTTCTCATTTCCACGATTTCCCAGAGAA |
| *Gapdh* | forward | CCAAGGAGTAAGAAACCCTGGACCACCC |
|  | reverse | TGTGAAGCTCATTTCCTGGTATGA |
|  | taq | CTCTCTTGCTCTCAGTATCCTTGCT |
| *Leptin* | forward | TCACCAGGATCAATGACATTTCAC |
|  | reverse | AGCCCAGGAATGAAGTCCAA |
|  | taq | ACGCAGTCGGTATCCGCCAAGC |
| *Gadd45a* | forward | TGAGCTGCTGCTACTGGAGAAC |
|  | reverse | CCGGCAAAAACAAATAAGTTGAC |
|  | taq | TGTGCTGGTGACGAACCCACATTCA |
| *bActin* | forward | TGACAGGATGCAGAAGGAGATTACT |
|  | reverse | GCCACCGATCCACACAGAGT |
|  | taq | ATCAAGATCATTGCTCCTCCTGAGCGC |
| *Socs3* | forward | CCACCCTCCAGCATCTTTGT |
|  | reverse | TCCAGGAACTCCCGAATGG |
|  | taq | ACTGTCAACGGCCACCTGGACTCCT |
| SYBR primer | | |
| *Nppa* | forward | CCTCGTCTTGGCCTTTTGG |
|  | reverse | GGTGGTCTAGCAGGTTCTTGAAA |
| *Faslg* | forward | CCATGGAGCGTATATGTGAACAG |
|  | reverse | AATGCCCACGTCACCAATG |
| *Gapdh* | forward | CCCCTTCATTGACCTCAACTACA |
|  | reverse | GGATCTCGCTCCTGGAAGATG |
| *Foxo3* | forward | ATGCTTCGCAACGATCCAA |
|  | reverse | TGGTGGAGCAAGTTCTGATTGA |
| *Foxo4* | forward | GACCACTTTCCGTCCACGAA |
|  | reverse | ACTTCAGACTCCGGCCTCATT |
| *Foxo6* | forward | TCCTACGCCGACCTCATCAC |
|  | reverse | CGGACCATCCAGTCGTAGATC |
| *Adiponectin* | forward | GACAAGGCCGTTCTCTTCAC |
|  | reverse | CCATACACCTGGAGCCAGAC |
| *Il1b* | forward | TGACAGTGATGAGAATGACCTGTTC |
|  | reverse | GGACAGCCCAGGTCAAAGG |
