# Supplementary material for: Maternal and perinatal obesity induce bronchial obstruction and pulmonary hypertension via IL-6-FoxO1-axis in later life
Source: Nat Commun. 2022 Jul 27;13:4352. doi: 10.1038/s41467-022-31655-z (PMC9329333; doi:10.1038/s41467-022-31655-z)
Supplement: Supplementary file 3 — Reporting Summary [file 41467_2022_31655_MOESM3_ESM.pdf]

nature research

Corresponding author(s): Miguel Angel Alejandre Alcázar, M.D., Ph.D.

Last updated by author(s): 2022-04-07

Reporting Summary

Nature Research wishes to improve the reproducibility of the work that we publish. This form provides structure for consistency and transparency in reporting. For further information on Nature Research policies, see Authors & Referees and the Editorial Policy Checklist .

Statistics

For all statistical analyses, confirm that the following items are present in the figure legend, table legend, main text, or Methods section.

n/a	Confirmed	
☐	☒	The exact sample size (*n*) for each experimental group/condition, given as a discrete number and unit of measurement
☐	☒	A statement on whether measurements were taken from distinct samples or whether the same sample was measured repeatedly
☐	☒	The statistical test(s) used AND whether they are one- or two-sided<br>*Only common tests should be described solely by name; describe more complex techniques in the Methods section.*
☒	☐	A description of all covariates tested
☐	☒	A description of any assumptions or corrections, such as tests of normality and adjustment for multiple comparisons
☐	☒	A full description of the statistical parameters including central tendency (e.g. means) or other basic estimates (e.g. regression coefficient) AND variation (e.g. standard deviation) or associated estimates of uncertainty (e.g. confidence intervals)
☐	☒	For null hypothesis testing, the test statistic (e.g. *F*, *t*, *r*) with confidence intervals, effect sizes, degrees of freedom and *P* value noted<br>*Give P values as exact values whenever suitable.*
☒	☐	For Bayesian analysis, information on the choice of priors and Markov chain Monte Carlo settings
☒	☐	For hierarchical and complex designs, identification of the appropriate level for tests and full reporting of outcomes
☒	☐	Estimates of effect sizes (e.g. Cohen's *d*, Pearson's *r*), indicating how they were calculated

Our web collection on statistics for biologists contains articles on many of the points above.

Software and code

Policy information about availability of computer code

Data collection	We used human data collected at the UK Biobank assessment centers (http://biobank.ctsu.ox.ac.uk/crystal/)
Data analysis	Raw fastq files were trimmed using BBduk and mapped to the Mus musculus genome (GhCm38, using gene annotation v97) STAR aligner according to Lexogene recommendations. Sequence reads were assigned to genomic features using featurecounts and DeSeq2 was used for statistical evaluation of significant differentially expressed genes. Plots were created using pheatmap and heatmaply and manhattanly for interactive volcano plots, for gene ontology enrichment analysis and pathway discovery was performed with clusterProfiler, gene annotation was performed with BioMart. Intron-spanning human and murine primers were designed using NCBI and UCSC Genome Browser database. CiiiDER softwarewas used as a tool for predicting transcription factor binding sites. Densitometric analysis of protein bands was performed using Bio-Rad ImageLab software Version 5.2.1 (Bio-Rad, Munich, Germany). Cell^D software Version 3.4 (Olympus) was applied for quantitatvie histomorphometric measurements. Statistical analyses were performed using the GraphPad Prism 7 Software. Photomicrographs of representative images were taken using a microscope (Olympus BX43, Hamburg, Germany) and using CellSens Dimension software (Version 1.18, Olympus).

For manuscripts utilizing custom algorithms or software that are central to the research but not yet described in published literature, software must be made available to editors/reviewers. We strongly encourage code deposition in a community repository (e.g. GitHub). See the Nature Research guidelines for submitting code & software for further information.

Data

Policy information about availability of data

All manuscripts must include a data availability statement. This statement should provide the following information, where applicable:

- Accession codes, unique identifiers, or web links for publicly available datasets
- A list of figures that have associated raw data
- A description of any restrictions on data availability

The RNA sequencing data has been deposited in Dryad (datadryad.org) with the accession number: https://doi.org/10.5061/dryad.zcrjdfnfh. All other data supporting the findings of this study are available within the article and its Supplementary Files. This research has been conducted using the UK Biobank Resource under Application Number 13721. We used human data collected at the UK Biobank assessment centers (http://biobank.ctsu.ox.ac.uk/crystal/) field ID 20258 and field ID 1687. Source data are provided with this paper.

Field-specific reporting

Please select the one below that is the best fit for your research. If you are not sure, read the appropriate sections before making your selection.

☒ Life sciences ☐ Behavioural & social sciences ☐ Ecological, evolutionary & environmental sciences

For a reference copy of the document with all sections, see nature.com/documents/nr-reporting-summary-flat.pdf

Life sciences study design

All studies must disclose on these points even when the disclosure is negative.

Sample size	The sample size was chosen based on prior studies from our showing sufficient statistical power for cell culture and animal experiments.
Data exclusions	Data were only exluded based on technical and experimental reasons or on calculation of a significant outlier using GraphPad Prism Software
Replication	to replicate the data animals from different litters were used. The data were reproducible.
Randomization	Animals were allocated to their respective group at birth by a blinded investigator.
Blinding	Investigators were blinded regarding experimental group (genetic modification, gene deletion).

Reporting for specific materials, systems and methods

We require information from authors about some types of materials, experimental systems and methods used in many studies. Here, indicate whether each material, system or method listed is relevant to your study. If you are not sure if a list item applies to your research, read the appropriate section before selecting a response.

Materials & experimental systems

n/a	Involved in the study	
☐	☒	Antibodies
☐	☒	Eukaryotic cell lines
☒	☐	Palaeontology
☐	☒	Animals and other organisms
☐	☒	Human research participants
☒	☐	Clinical data

Methods

n/a	Involved in the study	
☒	☐	ChIP-seq
☒	☐	Flow cytometry
☒	☐	MRI-based neuroimaging

Antibodies

Antibodies used

Immunoblot: mouse monoclonal anti-αSMA (Santa Cruz, #sc53142, clone: B4, 1:200, Dallas, TX, USA), monoclonal rabbit anti-pAKT (Cell signaling, #4058, clone 192H12, 1:1000, Danvers, MA, USA), polyclonal rabbit anti-AKT (Cell signaling, #9272; 1:2000, Danvers, MA, USA), polyclonal rabbit anti-pSAP/JNK1 (Thr183/Thr185; Cell signaling, #9251; 1:500, Danvers, MA, USA), polyclonal rabbit anti-SAP/JNK (Cell signaling, #9252; 1:1000, Danvers, MA, USA), monoclonal rabbit anti-p-c-Jun (Ser73; Cell signaling, #3270; clone D47G9, 1:1000, Danvers, MA, USA), monoclonal rabbit anti-c-Jun (Cell signaling, #9165; clone 60A8;1:1000 Danvers, MA, USA), polyclonal rabbit anti-PPARγ (Cell signaling, #2430; 1:1000, Danvers, MA, USA), polyclonal rabbit anti-pSTAT3 (Cell signaling, #9145; 1:1000, Danvers, MA, USA), polyclonal rabbit anti-STAT3 (Cell signaling, #9139; 1:1000, Danvers, MA, USA), polyclonal rabbit anti-p38 (Cell signalling, #9212, 1:2000, 1:1000, Danvers, MA, USA), monoclonal rabbit anti-pP38 (Cell signalling, #4511, clone: B3F9, 1:1000, 1:1000, Danvers, MA, USA), whereas monoclonal mouse-anti-β-Actin (Cell signaling, #3700, 1:5000, Danvers, MA, USA) served as a loading control. Anti-mouse IgG, HRP-linked (Cell signaling, # 7076, 1:2000, Danvers, MA, USA), and anti-rabbit IgG, HRP-linked (Cell signaling, # 7074, 1:2000, Danvers, MA, USA)
Immunostaining: polyclonal rabbit anti-FoxO1 (Novusbio, #NB100-2312, 1:400, Centennial, CO, USA), polyclonal rabbit anti-Ki67 (Thermo Fisher Scientific, #PAS19462, 1:1000, Waltham, MA, USA), polyclonal rabbit anti-pSTAT3 (Cell signaling, #9145; 1:200, Danvers, MA, USA), monoclonal rabbit anti-pAKT (Cell signaling, #4058, clone 193H12, 1:200, Danvers, MA, USA), p Histone H2A.X (S139, γH2AX; Cell signaling, #2577, 1:400, Danvers, MA, USA), monoclonal mouse anit-8-Oxo-dG (Abcam, #ab62623, clone 15A3, 1:400, Cambridge, UK) diluted in antibody diluent (DAKO, #S0809, Santa Clara, CA, United States), monoclonal-Cy3-anti-αSMA (Sigma Aldrich, #C6198, clone 1A4, 1:200, St. Louis, MO, USA), goat-raised secondary antibody, conjugated with an F488 fluorochrome (Jackson Laboratory, #111-485-003, 1:500, Bar Harbor, ME, USA)
Immunhistochemistry: monoclonal mouse anti-αSMA (Santa Cruz, # SC53142, clone: B4 1:200, St. Louis, MO, USA), polyclonal rabbit anti-von Willebrand factor (vWF; Abcam, #ab6994, 1:200, Cambridge, UK), polyclonal rabbit anti-CD68 (Abcam, #ab125212, 1:500, Cambridge, UK), monoclonal rabbit anti-CD3 (Thermo Fisher Scientific, #RM-9107-S, Clone SP7, 1:50, Waltham, MA, USA), monoclonal rat anti-CD45 (BD, #550539, Clone 30-F11, 1:25, Franklin Lakes, USA) or monoclonal rat anti-LyG6 (Abcam, #ab2557, 1:50, Cambridge, UK).

Validation

Antibodies used in this manuscript are validated by the respective manufacturer.

Eukaryotic cell lines

Policy information about cell lines

Cell line source(s)	Human bronchial SMC (hbSMC) were purchased from PromoCell (#397Z013.3, #C-12561, Heidelberg, Germany); primary murine tracheal smooth muscle cells were isolated from young mice.
Authentication	Cells were stained for alpha SMA.
Mycoplasma contamination	Cells were tested for mycoplasma contamination
Commonly misidentified lines (See ICLAC register)	n/a

Palaeontology

Specimen provenance

Provide provenance information for specimens and describe permits that were obtained for the work (including the name of the issuing authority, the date of issue, and any identifying information).

Specimen deposition

Indicate where the specimens have been deposited to permit free access by other researchers.

Dating methods

If new dates are provided, describe how they were obtained (e.g. collection, storage, sample pretreatment and measurement), where they were obtained (i.e. lab name), the calibration program and the protocol for quality assurance OR state that no new dates are provided.

☐ Tick this box to confirm that the raw and calibrated dates are available in the paper or in Supplementary Information.

Animals and other organisms

Policy information about studies involving animals; ARRIVE guidelines recommended for reporting animal research

Laboratory animals

C57BL/6 (WT), B6.129S2IL-6tm1Kopf/J, Mice with a smooth muscle cell-specific deletion of Foxo1 were generated by crossing mice homozygous for the floxed Foxo1 allele with Sm22-cre mice (Tagln-cre 1Her/J, the Jackson Laboratory, Bar Harbor, ME). The offspring, Foxo1flox/flox-Sm22-cre (SMCFoxo1-KO), and their littermates, control Foxo1+/+-Sm22-cre (WT), were used for experiments. $FoxO1^{ADA\text{-}fl/fl}$ were kindly provided by Dr. Thomas Wunderlich (Max Planck Institute for Metabolic Research, University Hospital Cologne, Cologne, Germany)Only male offspring at day P21 or P70 have been used in this study.

Wild animals

no wild animals were used in this study

Field-collected samples

no field-collected samples were used in this study

Ethics oversight

The experiments were performed in accordance with German regulations and legal requirements and were approved by the local government authorities (LANUV, NRW, Germany; AZ # 2018A320, AZ # 2012A424, AZ # 2011-025, and AZ# 50.15.015; Regierungspräsidium Giessen and Darmstadt, Hessen, Germany; approval numbers B2/318; B2/311).

Note that full information on the approval of the study protocol must also be provided in the manuscript.

Human research participants

Policy information about studies involving human research participants

Population characteristics

The UK Biobank is a longitudinal cohort study of >500,000 individuals aged 40 to 69 years initiated in the United Kingdom in 2006–2010. The exposures of interest for our main analysis were forced expiratory volume in 1 s/forced vital capacity (FEV1/FVC) ratio Z-score (field ID 20258) and comparative body size at 10 years of age (field ID 1687, recoded into two groups: about average and thinner = 0; plumper = 1). For our analyses, we excluded 1,756 individuals due to Chronic Obstructive Pulmonary Disease (COPD), and 52,990 current smokers. After these exclusions, 448,351 individuals were eligible for observational analyses. The exposures of interest for our main analysis were forced expiratory volume in 1 s/forced vital capacity (FEV1/FVC) ratio Z-score (field ID 20258), and comparative body size at 10 years of age (field ID 1687, recoded to two groups: about average and thinner=0; plumper =1). Asthma was defined as International Classification of Diseases (ICD) edition 10 codes J45.

Recruitment

We used data collected at the UK Biobank assessment centers at baseline, combined with information collected in the hospital (http://biobank.ctsu.ox.ac.uk/crystal/).

Ethics oversight

https://www.ukbiobank.ac.uk/ethics/
This research has been conducted using the UK Biobank Resource under Application Number 13721.

Note that full information on the approval of the study protocol must also be provided in the manuscript.

Clinical data

Policy information about clinical studies

All manuscripts should comply with the ICMJEguidelines for publication of clinical research and a completedCONSORT checklist must be included with all submissions.

Clinical trial registration

Provide the trial registration number from ClinicalTrials.gov or an equivalent agency.

Study protocol

Note where the full trial protocol can be accessed OR if not available, explain why.

Data collection

Describe the settings and locales of data collection, noting the time periods of recruitment and data collection.

Outcomes

Describe how you pre-defined primary and secondary outcome measures and how you assessed these measures.

ChIP-seq

Data deposition

☐ Confirm that both raw and final processed data have been deposited in a public database such as GEO.

☐ Confirm that you have deposited or provided access to graph files (e.g. BED files) for the called peaks.

Data access links<br>*May remain private before publication.*	*For "Initial submission" or "Revised version" documents, provide reviewer access links. For your "Final submission" document, provide a link to the deposited data.*
Files in database submission	*Provide a list of all files available in the database submission.*
Genome browser session<br>(e.g. UCSC)	*Provide a link to an anonymized genome browser session for "Initial submission" and "Revised version" documents only, to enable peer review. Write "no longer applicable" for "Final submission" documents.*

Methodology

Replicates	*Describe the experimental replicates, specifying number, type and replicate agreement.*
Sequencing depth	*Describe the sequencing depth for each experiment, providing the total number of reads, uniquely mapped reads, length of reads and whether they were paired- or single-end.*
Antibodies	*Describe the antibodies used for the ChIP-seq experiments; as applicable, provide supplier name, catalog number, clone name, and lot number.*
Peak calling parameters	*Specify the command line program and parameters used for read mapping and peak calling, including the ChIP, control and index files used.*
Data quality	*Describe the methods used to ensure data quality in full detail, including how many peaks are at FDR 5% and above 5-fold enrichment.*
Software	*Describe the software used to collect and analyze the ChIP-seq data. For custom code that has been deposited into a community repository, provide accession details.*

Flow Cytometry

Plots

Confirm that:

☐ The axis labels state the marker and fluorochrome used (e.g. CD4-FITC).

☐ The axis scales are clearly visible. Include numbers along axes only for bottom left plot of group (a 'group' is an analysis of identical markers).

☐ All plots are contour plots with outliers or pseudocolor plots.

☐ A numerical value for number of cells or percentage (with statistics) is provided.

Methodology

Sample preparation	*Describe the sample preparation, detailing the biological source of the cells and any tissue processing steps used.*
Instrument	*Identify the instrument used for data collection, specifying make and model number.*
Software	*Describe the software used to collect and analyze the flow cytometry data. For custom code that has been deposited into a community repository, provide accession details.*
Cell population abundance	*Describe the abundance of the relevant cell populations within post-sort fractions, providing details on the purity of the samples and how it was determined.*
Gating strategy	*Describe the gating strategy used for all relevant experiments, specifying the preliminary FSC/SSC gates of the starting cell population, indicating where boundaries between "positive" and "negative" staining cell populations are defined.*

☐ Tick this box to confirm that a figure exemplifying the gating strategy is provided in the Supplementary Information.

Magnetic resonance imaging

Experimental design

Design type	*Indicate task or resting state; event-related or block design.*

Design specifications | *Specify the number of blocks, trials or experimental units per session and/or subject, and specify the length of each trial or block (if trials are blocked) and interval between trials.*

Behavioral performance measures | *State number and/or type of variables recorded (e.g. correct button press, response time) and what statistics were used to establish that the subjects were performing the task as expected (e.g. mean, range, and/or standard deviation across subjects).*

Acquisition

Imaging type(s) | *Specify: functional, structural, diffusion, perfusion.*

Field strength | *Specify in Tesla*

Sequence & imaging parameters | *Specify the pulse sequence type (gradient echo, spin echo, etc.), imaging type (EPI, spiral, etc.), field of view, matrix size, slice thickness, orientation and TE/TR/flip angle.*

Area of acquisition | *State whether a whole brain scan was used OR define the area of acquisition, describing how the region was determined.*

Diffusion MRI ☐ Used ☒ Not used

Preprocessing

Preprocessing software | *Provide detail on software version and revision number and on specific parameters (model/functions, brain extraction, segmentation, smoothing kernel size, etc.).*

Normalization | *If data were normalized/standardized, describe the approach(es): specify linear or non-linear and define image types used for transformation OR indicate that data were not normalized and explain rationale for lack of normalization.*

Normalization template | *Describe the template used for normalization/transformation, specifying subject space or group standardized space (e.g. original Talairach, MNI305, ICBM152) OR indicate that the data were not normalized.*

Noise and artifact removal | *Describe your procedure(s) for artifact and structured noise removal, specifying motion parameters, tissue signals and physiological signals (heart rate, respiration).*

Volume censoring | *Define your software and/or method and criteria for volume censoring, and state the extent of such censoring.*

Statistical modeling & inference

Model type and settings | *Specify type (mass univariate, multivariate, RSA, predictive, etc.) and describe essential details of the model at the first and second levels (e.g. fixed, random or mixed effects; drift or auto-correlation).*

Effect(s) tested | *Define precise effect in terms of the task or stimulus conditions instead of psychological concepts and indicate whether ANOVA or factorial designs were used.*

Specify type of analysis: ☐ Whole brain ☐ ROI-based ☐ Both

Statistic type for inference
(See Eklund et al. 2016) | *Specify voxel-wise or cluster-wise and report all relevant parameters for cluster-wise methods.*

Correction | *Describe the type of correction and how it is obtained for multiple comparisons (e.g. FWE, FDR, permutation or Monte Carlo).*

Models & analysis

n/a	Involved in the study
☒	☐ Functional and/or effective connectivity
☒	☐ Graph analysis
☒	☐ Multivariate modeling or predictive analysis
